# Supplementary material for: Association of family structure with gain and loss of household headship among older adults in India: Analysis of panel data
Source: PLoS One. 2021 Jun 4;16(6):e0252722. doi: 10.1371/journal.pone.0252722 (PMC8177662; doi:10.1371/journal.pone.0252722)
Supplement: Attachment — Submitted filename: review report.docx [file pone.0252722.s001.docx]

Review: Association of Family Structure with Gain and Loss of Household Headship among Older Adults of India: Analysis of Panel Data

This article addresses the important topic the association of family structure with gain and loss of household headship among older adults of India from the panel data of IHDS wave-I and wave-II. The outcome indicators for the study was the gain in headship and loss in headship with other explanatory and control variables. Authors have concluded that the loss of household headship was significantly associated with the changes in the family structure. The results of the multivariate analysis found that older adults from economically poor background and older adults with chronic disease conditions had higher chances of losing headship. Although, the paper has some limitations. Here some broad suggestions:

1. Page no. 8 (208-209) Authors have mentioned that they have found the interaction effect of marital status, income, education level and household BPL status. But, did not find any explanation behind it. Authors would have added table for the changes in the characteristics of older adults in both rounds of data sets which are directly affecting the household headship status like marital status, working status and Household BPL status. The interaction effect of these variables can be looked up. The work status and marital status decides the living arrangement of older adults, and living arrangement affects the household headship status. If any older adults who was not in poor in wave-I but entered in poor (BPL) category or vice-versa, this can affect the work status of the older adults.
2. Page no. 7 (164-167). The categorization of marital status variable is not clear. It can be written in more clear way.
3. Page no. 17-18. In the discussion section, culture of the Indian society can be discussed more in term of voluntary transfer of headship to their economically active sons.

Minor comment

1. Page no. 16. ® Symbol can be assigned against the reference category in the table 3 as mentioned in note.
