# Supplementary material for: Association of family structure with gain and loss of household headship among older adults in India: Analysis of panel data
Source: PLoS One. 2021 Jun 4;16(6):e0252722. doi: 10.1371/journal.pone.0252722 (PMC8177662; doi:10.1371/journal.pone.0252722)
Supplement: Attachment — Submitted filename: Response to Reviewers.docx [file pone.0252722.s002.docx]

**MANUSCRIPT TITLE:** Association of Family Structure with Gain and Loss of Household Headship among Older Adults of India: Analysis of Panel Data

**SUBJECT:** Response to reviewers

Dear editor,

Thank you for giving us the opportunity of submitting an improved version of our manuscript for publication in the PLOS One. We are highly grateful to you and both the reviewers for their insightful comments and suggestions. We appreciate the time and effort that you and the reviewers have put forward to provide valuable feedback that has significantly improved our paper. Kindly note that we have incorporated all of the changes that were suggested. The modifications have been shown using track changes within the revised manuscript. Please see below for a point-by-point response to each of the comments and suggestions.

Hoping that you and your family members are safe and sound in these challenging times,

Yours Sincerely,

Authors

**Journal Requirements**

1. In your ethics statement in the Methods section and the online submission form, please provide additional information about the data used in your retrospective study. Specifically, please clarify whether all data were fully anonymized before you accessed them."

Response: Thank you for the suggestion. Accordingly, we have the following statement in the “Ethics statement” section of the manuscript (page 5, line 120-122):

“Our study utilized publicly available secondary datasets with no information that could lead to identification of the respondents. Therefore, prior ethical approval for using these datasets were not necessary.”

The same has also been updated in the online submission form.

2. Please provide the numerical p-values in Tables 2 and 3.

Response: In line with the editor’s suggestion, numerical p-values have been provided in Tables 2 and 3.

3. Please include the date(s) on which you accessed the databases or records to obtain the retrospective data used in your study.

Response: Thank you for pointing this out. Accordingly, the date of accessing the databases have been provided in the Reference to the datasets (Page 24, line 454-460).

**Reviewer’s Comment**

**Reviewer #1:** This article addresses the important topic the association of family structure with gain and loss of household headship among older adults of India from the panel data of IHDS wave-I and wave-II. The outcome indicators for the study was the gain in headship and loss in headship with other explanatory and control variables. Authors have concluded that the loss of household headship was significantly associated with the changes in the family structure. The results of the multivariate analysis found that older adults from economically poor background and older adults with chronic disease conditions had higher chances of losing headship. Although, the paper has some limitations. Here some broad suggestions:

1. Page no. 8 (208-209) Authors have mentioned that they have found the interaction effect of marital status, income, education level and household BPL status. But, did not find any explanation behind it. Authors would have added table for the changes in the characteristics of older adults in both rounds of data sets which are directly affecting the household headship status like marital status, working status and Household BPL status. The interaction effect of these variables can be looked up. The work status and marital status decides the living arrangement of older adults, and living arrangement affects the household headship status. If any older adults who was not in poor in wave-I but entered in poor (BPL) category or vice-versa, this can affect the work status of the older adults.

Response: Dear reviewer, thank you very much for pointing this out. However, keeping in mind the comment given by the editor and reviewer #2 we have refrained from examining interaction effects further. However, we have included this point as a scope for further research in the “Discussion” section (Page 20, line 380-382).

2. Page no. 7 (164-167). The categorization of marital status variable is not clear. It can be written in more clear way.

Response: Thank you for the suggestion. Accordingly, we have modified the line to (Page 7, line 167-169):

*“Individuals who were “single”, “widowed”, “separated/divorced”, “spouse absent” and “no gauna” were recoded into the “Not currently married” category; otherwise, they were included in the “Currently married” category.”*

3. Page no. 17-18. In the discussion section, culture of the Indian society can be discussed more in term of voluntary transfer of headship to their economically active sons.

Response: comment incorporated (Line no. 323-327)

Minor comment

1. Page no. 16. ® Symbol can be assigned against the reference category in the table 3 as mentioned in note.

Response: Thank you very much for pointing this out. We have denoted the reference category in Table 3 by assigning the symbol (Ref).

**Reviewer #2:** The authors conclude that the loss or gain of headship was associated with changes in the family structure but their analysis does not reflect this. The multivariate results primarily show the odds of gaining or losing headship status given that one belongs to a particular family type but do not analyze whether the gain or loss can be attributed to a change in family type. For instance, the older person might have lost headship due to a change from nuclear to joint/extended family.

Response: Dear sir, Thank you for the very important insight. I completely agree with your comment. I have rephrased the first line of conclusion. (Line no. 388-389)

1. It is unclear the reason for using characteristics in wave I when these might have changed in wave II. Maybe it is an issue with how it has been conceptualized. Can authors explain this?

Response: We agree that characteristics such as marital status, household wealth quintile, and household BPL status might change between round-I and round-II. However, our objective is to check whether the characteristics in round-I were associated with the change in household headship status between round-II. Accordingly, the study has been conceptualized. In the present study we just tried to find the predictors of loss or gain of headship among older adults.

2. For the multivariate analysis, authors could consider running two models for each of the outcomes. The first model could contain only the primary factors - family type - since it is the main association authors wish to test. The second model will then account for the effect of control variables. The odds ratio and CI can be reported in one column (in order to get enough space for the two models). Also, authors could indicate in the table the reference category for the independent variables as Ref or 1.00. Using ® (which did not appear at all in table 3) is somehow non-academic. In table 3, square brackets should be used instead when reporting CIs. Also, authors should use a comma instead of "dash". e.g. [0.67, 1.40]

Response: We agree with the reviewer’s suggestion. Therefore, keeping in mind the editor’s and the reviewer’s suggestion regarding formatting of Table 3 we have denoted CI within square brackets separated by a comma in between. Further, reference category for independent variables has been shown using Ref.

As per your suggestion, we considered running two separate models with the first model showing the bivariate association of family type in round-I with headship change between round-I and round-II and the second model accounting for the effect of control variables. We humbly point out that bivariate association has of family type in round-I with headship change between round-I and round-II has already been examined in Table 2. Evidently, we did not repeat the same in Table 3.

3. Under the statistical methods, can authors explain their reason for testing interactions? in my opinion, there may not be a need to write on interactions. This is because the objective of this study was not to investigate the interactions between specific variables.

Response: Dear reviewer, thank you for pointing this out. Accordingly, we have omitted the section for testing of interactions in the revised manuscript.

4. line 227 - ... wave I increased instead of "were increased".

Response: Thank you for pointing this out. We have corrected the sentence to (Page 9, line 223-224):

*As shown in figure 2, the percentage of older adults who were residing in a single generation household in wave-I increased to nearly 6% in wave-II.*

5. line 236 - authors could consider using "no formal education or no formal schooling" instead of no schooling education.

Response: Thank you for pointing this out. We have corrected the sentence to (Page 10, line 232):

*Further, 67% of older adults were married, 60% had no formal education and 85% were unemployed during wave-I.*

6. Authors could also briefly explain how the limitations link to their study. For instance, how is the non-inclusion of the dependency status of the son a limitation for this study.

Response: Comment incorporated. (Line 375-386)
