# Supplementary material for: Association of family structure with gain and loss of household headship among older adults in India: Analysis of panel data
Source: PLoS One. 2021 Jun 4;16(6):e0252722. doi: 10.1371/journal.pone.0252722 (PMC8177662; doi:10.1371/journal.pone.0252722)
Supplement: Attachment — Submitted filename: Response to Reviewers.docx [file pone.0252722.s003.docx]

**MANUSCRIPT TITLE:** Association of Family Structure with Gain and Loss of Household Headship among Older Adults in India: Analysis of Panel Data

**SUBJECT:** Response to reviewers

Respected editor,

Thank you for giving us the opportunity of submitting an improved version of our manuscript for publication in the PLOS One. We are highly grateful to receive your insightful comments and suggestions. We appreciate the time and effort that you have put forward to provide valuable feedback that has significantly improved our paper. Kindly note that we have incorporated the changes that were suggested. The modifications have been shown using track changes within the revised manuscript. Please see below for a point-by-point response to each of the comments and suggestions.

Hoping that you and your family members are safe and sound in these challenging times,

Yours Sincerely,

Authors

**Editor’s Comments:**

1. Dear Authors, I have asked you to include some variables in the background characteristics: ‘the number of married, unmarried adults and children in the family. Without understanding the age and marital status composition of other household members, it is difficult to explain why and when headship transition takes place in families. In general, the marriage of children is the first break-point for older adult headship.

Response: Dear editor thank you for the suggestion. We have included variables showing the number of married adults, number of unmarried adults and number of children in the family, in our analysis. Accordingly, the “Data and methods”, “Results” and “Discussion” section and the tables have been updated.

2. What kind of headship loss you’re talking about for single-person older households, whom he/she is heading. What do you understand by headship? I think you need to put some logic while planning the analyses.

Response: Dear sir, we are not considering single-person older households. For the headship to be loosed, at least 2 persons should reside in a single household. Secondly, headship means the person who is heading the household, taking all the major decisions. In the large-scale survey, we generally ask all the household information from the household head only. Therefore, the dynamics of headship in IHDS-1 and IHDS-2 was observed from the present analysis.

3. Also, this paper is still silent on the question that what had happened between the waves that an older adult has to give up his/her headship? IHDS allows adding such variables.

Response: Dear editor, thank you for pointing this out. Kindly note that in this paper we aim to analyse the association of family structure and other explanatory variables in wave-I with their change in headship status between wave-I and wave-II. The same has also been pointed out by Reviewer #2. We do not try to answer the question that what had happened between the waves that an older adult has to give up his/her headship.

We know that using the “IHDS tracking sheet” we can determine whether an older adult died or was untraceable between round-I and round-II. Since we are using the panel data, it effectively means that we are analysing only those adults who were alive and traceable by IHDS staff after wave-I and during wave-II. Further, we have already noted the following in our manuscript (Page 5, line 114-117):

*During IHDS wave-I, there were 17,906 individuals, aged 60 years and above, whom we refer to as older adults in this study. Among them, 4,736 older adults were not alive and 2,643 older adults were lost to follow-up during wave-II. Thus, our current study is based on panel data of 10,527 older adults.*

4. Also, bring some sociological or family demography literature on why and when headship transition in the households takes place and whether your analyses are supporting or rejecting that hypotheses?

Response: Comment incorporated.

5. Also, can you cut down the length of headings in the manuscript and Tables?

Response: Thank you. We have shortened the length of headings in the manuscript and tables.

6. If you feel these suggestions are not relevant to your paper, please respond to them.

Response: Dear editor, on a contrary note we believe that all the suggestions given by you have improved our manuscript by leaps and bounds. Therefore, we have carefully studied, incorporated and then responded to each of them.
